# Supplementary material for: Deep learning for the prediction of early on-treatment response in metastatic colorectal cancer from serial medical imaging
Source: Nat Commun. 2021 Nov 17;12:6654. doi: 10.1038/s41467-021-26990-6 (PMC8599694; doi:10.1038/s41467-021-26990-6)
Supplement: Supplementary file 1 — Supplementary information. [file 41467_2021_26990_MOESM1_ESM.pdf]

# Supplementary Information

## Title: Deep learning for the prediction of early on-treatment response in metastatic colorectal cancer from serial medical imaging

# Content Index (Page Number)

### Supplementary Figures


Supplementary Figure 1. Kaplan-Meier analysis for the discovery and test cohorts. 2

Supplementary Figure 2. ROC of DL prediction score for the tuning set. 3

Supplementary Figure 3. The landmark analysis on the DL network starting on month-2. 4

Supplementary Figure 4. ROC of ETS for the tuning set. 5

Supplementary Figure 5. Linear correlation between DL prediction score and ETS 6

Supplementary Figure 6. Nomograms for the RECIST, TB, ETS, DL-BS, DL-PS and Size-Nomo models. 7

Supplementary Figure 7. Calibration curves for the RECIST, TB, ETS, DL-BS, DL-PS and Size-Nomo models in discovery cohort. 8

Supplementary Figure 8. Calibration curves for the RECIST, TB, ETS, DL-BS, DL-PS and Size-Nomo models in test cohort. 9

Supplementary Figure 9. The network structure of GoogLeNet 12

Supplementary Figure 10. An example for input of DL network on training 14


### Supplementary Tables


Supplementary Table 1. Summary of comparison between DL and ETS criteria 10


### Supplementary Methods


GoogLeNet Fine-tuning 12

ROI preparation 13

RNN construction and training 15

Reproducibility analysis 16


### Supplementary References


References 17

# Supplementary Figures

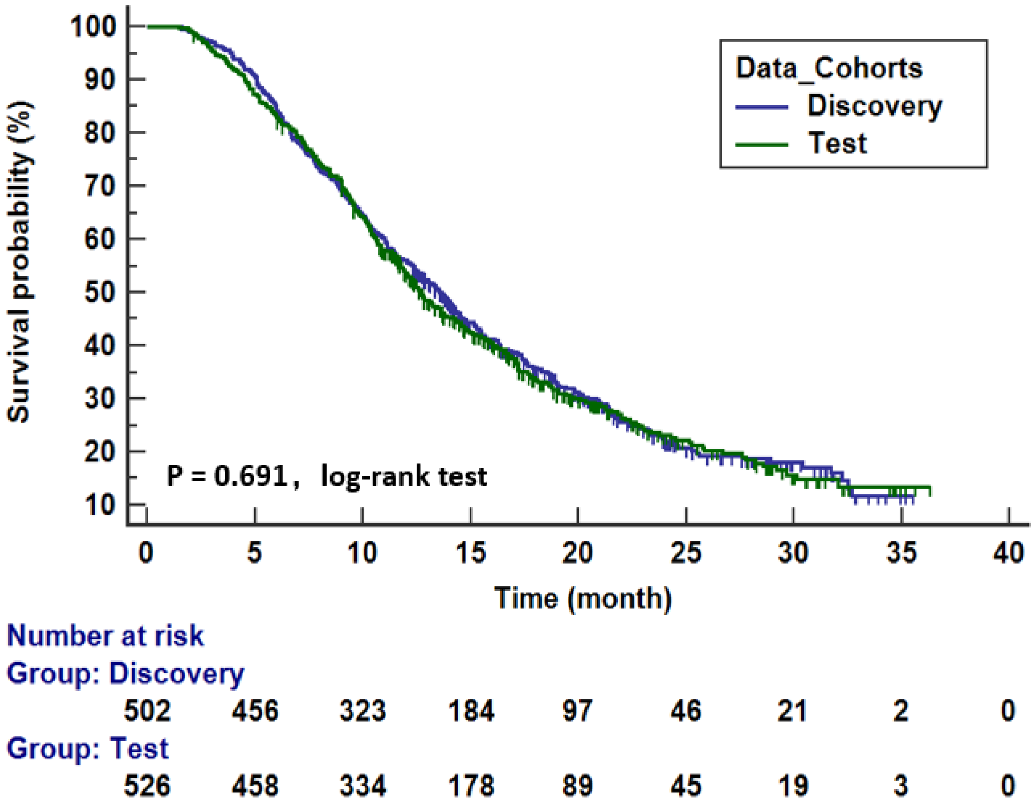


**Supplementary Figure 1. Kaplan-Meier analysis for the discovery and test cohorts.** The media OS for patients were 13.56 and 12.71 months in the discovery and test cohorts, respectively. There is no significant difference between the discovery and test cohorts (p = 0.691, log-rank test).

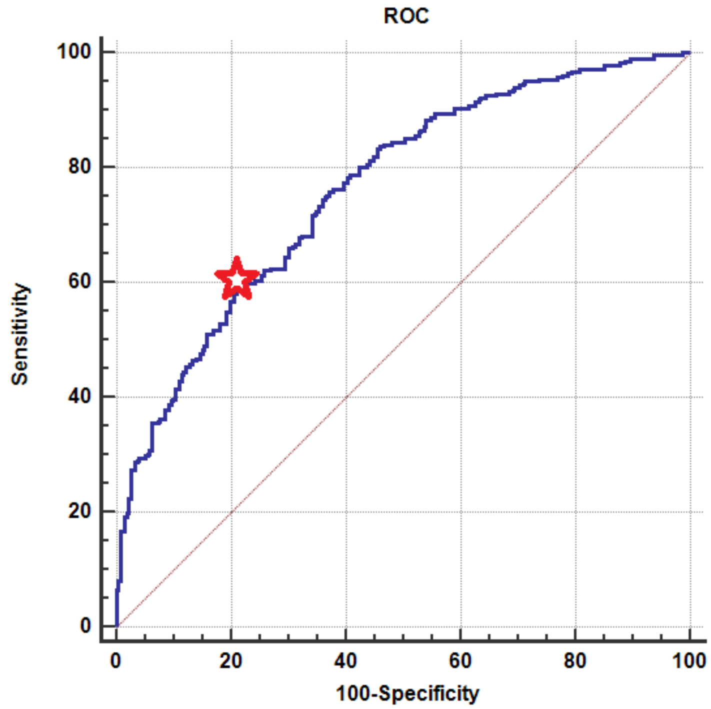


**Supplementary Figure 2. The ROC of DL prediction score on the tuning set.** The optimal point (indicated by the star) was found at ‘criterion: ≥0.6’, where the corresponding sensitivity and specificity were 60.6% and 78.6%, respectively.

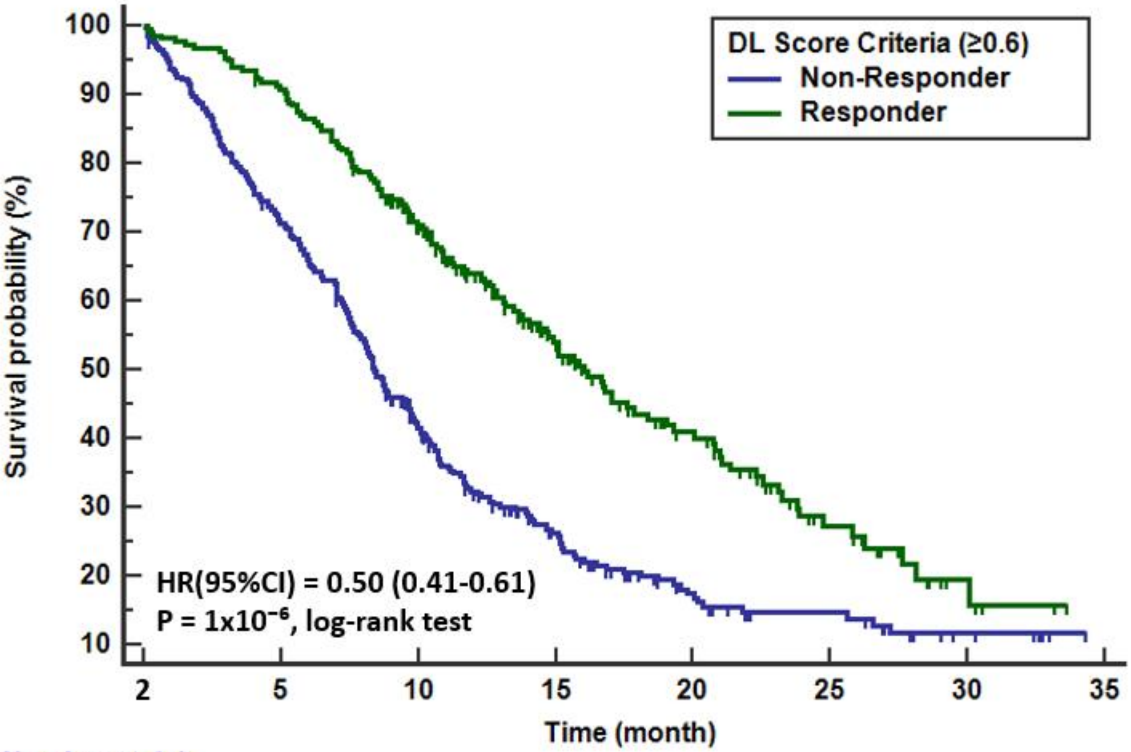


Number at risk

| Group | 2 | 5 | 10 | 15 | 20 | 25 | 30 | 35 |
|---|---|---|---|---|---|---|---|---|
| Group: Non-Responder | 310 | 219 | 116 | 58 | 27 | 16 | 6 | 0 |
| Group: Responder | 216 | 195 | 140 | 81 | 45 | 18 | 5 | 0 |

**Supplementary Figure 3. The landmark analysis on the DL network starting on month-2.**

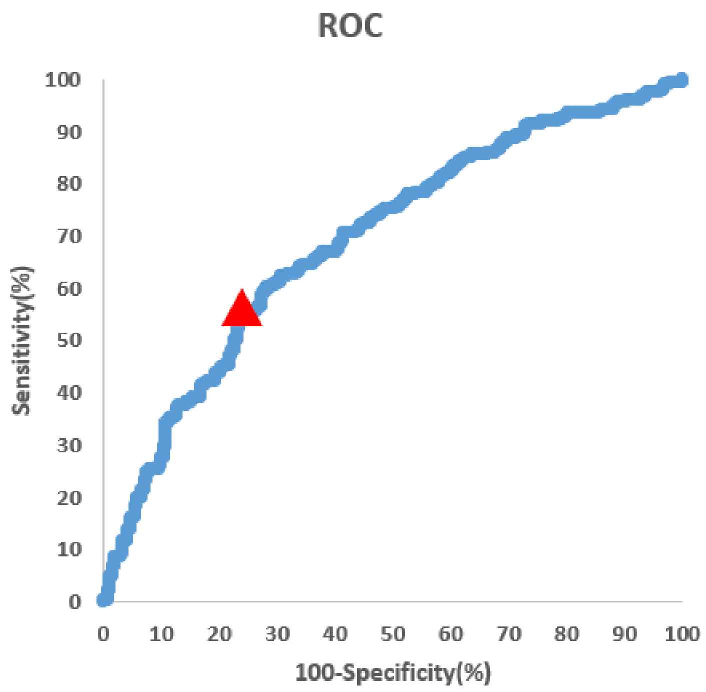


**Supplementary Figure 4. The ROC of ETS on the tuning set.** The optimal point (indicated by the star) was found at ‘criterion: ≥0.05’, where the corresponding sensitivity and specificity were 59.5% and 72.3, respectively.

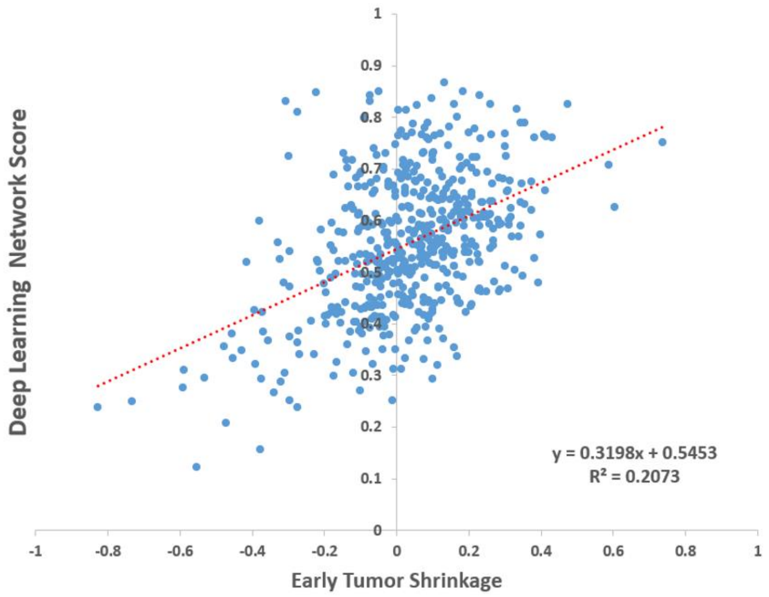


**Supplementary Figure 5. Linear correlation between DL prediction score and ETS.**

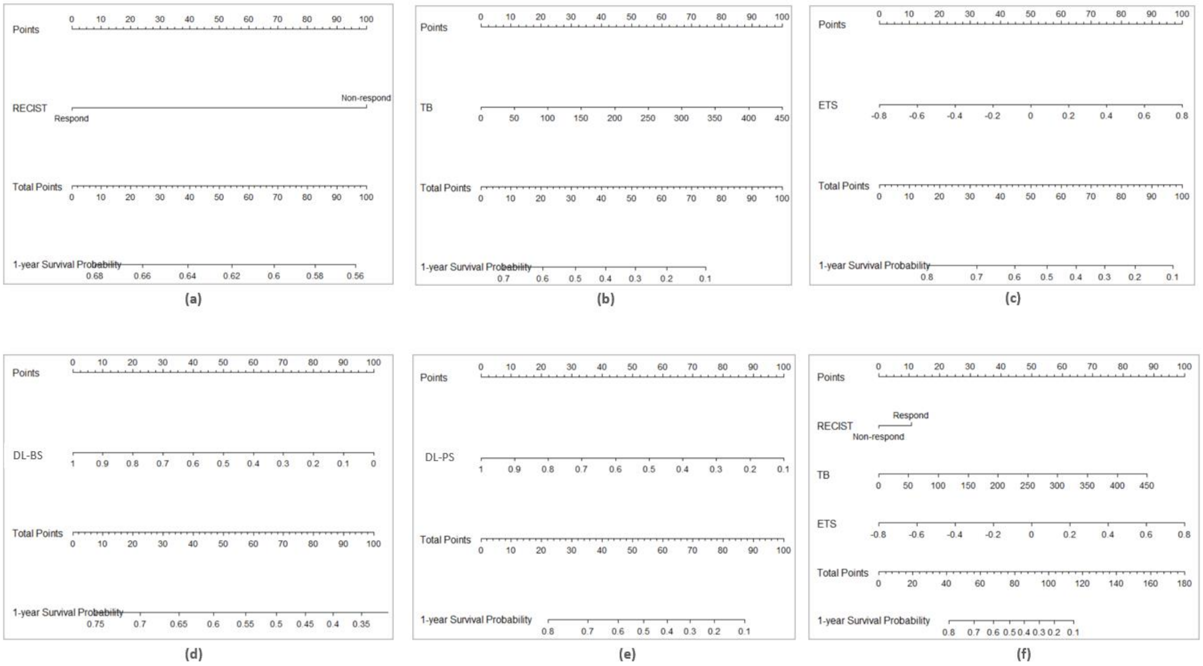


**Supplementary Figure 6. (a-f) are the nomograms for the RECIST, TB, ETS, DL-BS, DL-PS and Size-Nomo models, respectively.**

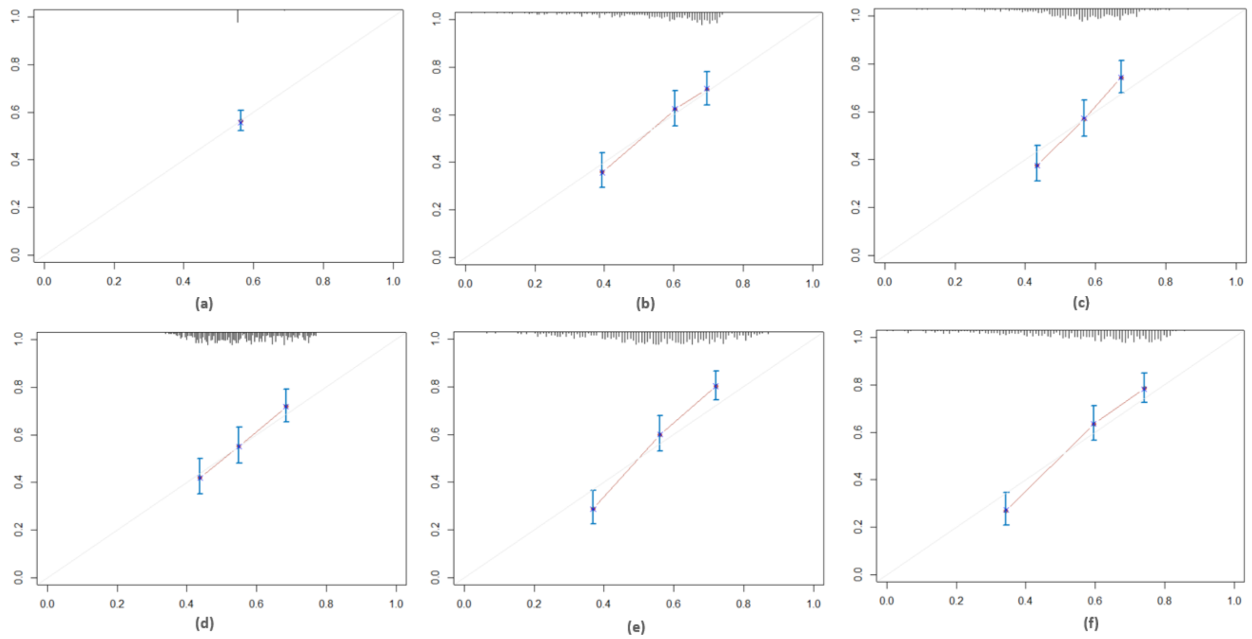


**Supplementary Figure 7. (a-f) are the calibration curves in the discovery cohort (n = 502 patients) for the RECIST, TB, ETS, DL-BS, DL-PS and Size-Nomo models, respectively. The predicted survival probability for the three patient groups (The short, median, and long survival groups with patients of n = 502/3 in each group) were shown as error bars, i.e., mean value ± standard error.**

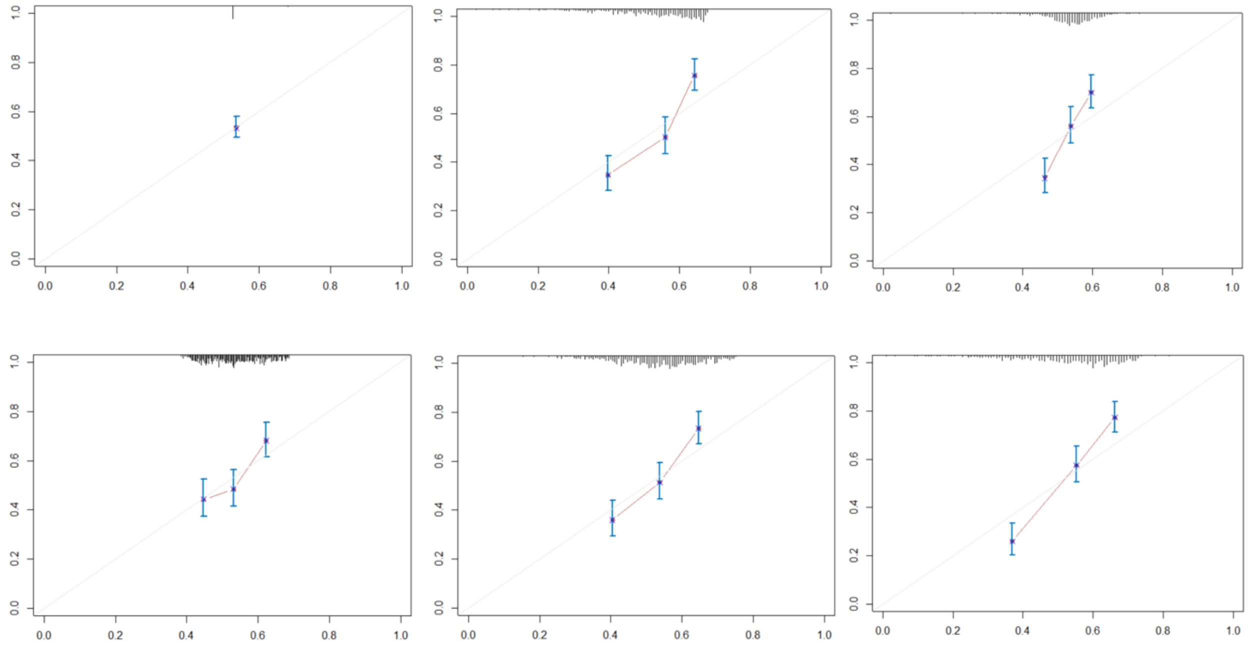

**Supplementary Figure 8. (a-f) are the calibration curves in the discovery cohort (n = 526 patients) for the RECIST, TB, ETS, DL-BS, DL-PS and Size-Nomo models, respectively. The predicted survival probability for the three patient groups (The short, median, and long survival groups with patients of n = 526/3 in each group) were shown as error bars, i.e., mean value ± standard error.**

# Supplementary Table

## Supplementary Table 1. Summary of comparison between DL and ETS criteria.

| Therapy & Line | Trail & Assessment Criterion | Treatment Arms | Patient Number | Criterion Res vs. Non-res | Respond Rate / Not Respond Rate | | Median OS (months) | | | HR (95%CI) | p-value* |
|---|---|---|---|---|---|---|---|---|---|---|---|
| | | | | | Res | Non-res | Res | Non-res | Δ | | |
| Chemo. ± anti-VEGF in 2nd line | VELOUR with optimal DL criterion | FOLFIRI + Afl. | 259 | Score ≥ 0.6 vs. <0.6 | 46% | 55% | 18.1 | 10.8 | 7.3 | 0.56 (0.41-0.76) | <0.001 |
| | | FOLFIRI | 267 | Score ≥ 0.6 vs. <0.6 | 37% | 63% | 18 | 10.2 | 7.8 | 0.45 (0.34-0.60) | <0.001 |
| | VELOUR with optimal ETS criterion | FOLFIRI + Afl. | 259 | ETS ≥ 5% vs. <5% | 59% | 41% | 16 | 10.6 | 5.4 | 0.57 (0.41-0.79) | <0.001 |
| | | FOLFIRI | 267 | ETS ≥ 5% vs. <5% | 39% | 62% | 15.2 | 10.6 | 4.6 | 0.65 (0.49-0.86) | <0.001 |
| | VELOUR with ETS criterion 20% | FOLFIRI + Afl. | 259 | ETS ≥ 20% vs. <20% | 25% | 75% | 18.6 | 12 | 6.6 | 0.48 (0.35-0.67) | <0.001 |
| | | FOLFIRI | 267 | ETS ≥ 20% vs. <20% | 11% | 89% | 17.2 | 11.8 | 5.4 | 0.61(0.42-0.89) | <0.001 |
| | VELOUR with ETS criterion 30% | FOLFIRI + Afl. | 259 | ETS ≥ 30% vs. <30% | 10% | 90% | 15.5 | 12.7 | 2.8 | 0.71 (0.44-1.15) | NS |
| | | FOLFIRI | 267 | ETS ≥ 30% vs. <30% | 4% | 96% | 27.6 | 11.9 | 15.7 | 0.52 (0.30-0.91) | NS |
| Chemo. ± anti-EGFR in 2nd line | BOND with optimal ETS criterion | Cet. + irinotecan | 192 | ETS ≥ 10% vs. <10% | 37% | 63% | 12 | 7.4 | 4.6 | 0.33 (0.22-0.52) | <0.001 |
| | | Cet. | 97 | ETS ≥ 10% vs. <10% | 28% | 72% | na | na | na | na | na |
| | 20050181 study with ETS criterion 30% | FOLFIRI+Pani. | 181 | ETS ≥ 30% vs. <30% | 37% | 63% | na | na | na | na | na |
| | | FOLFIRI | 180 | ETS ≥ 30% vs. <30% | 7% | 93% | na | na | na | na | na |
| Chemo. ± anti-EGFR in 1st line | CRYSTAL with ETS criterion 20% | FOLFIRI + Cet. | 299 | ETS ≥ 20% vs. <20% | 62% | 38% | 30 | 18.6 | 11.4 | 0.53 (0.40-0.69) | <0.001 |
| | | FOLFIRI | 332 | ETS ≥ 20% vs. <20% | 49% | 51% | 24.1 | 18.6 | 5.5 | 0.71 (0.56-0.91) | 0.006 |
| | OPUS with ETS criterion 20% | FOLFOX-4 + Cet. | 78 | ETS ≥ 20% vs. <20% | 69% | 31% | 26 | 15.7 | 10.3 | 0.43 (0.23-0.78) | 0.006 |
| | | FOLFOX-4 | 90 | ETS ≥ 20% vs. <20% | 46% | 54% | 21.6 | 17.8 | 3.8 | 0.89 (0.53-1.47) | NS |
| | PRIME with ETS criterion 20% | FOLFOX-4 + Pani. | 219 | ETS ≥ 20% vs. <20% | 72% | 28% | 32.5 | 12.6 | 19.9 | 0.47 (0.34-0.65) | <0.001 |
| | | FOLFOX-4 | 221 | ETS ≥ 20% vs. <20% | 57% | 43% | 26 | 15.2 | 10.8 | 0.50 (0.37-0.66) | <0.001 |
| | PRIME with ETS criterion 30% | FOLFOX-4 + Pani. | 219 | ETS ≥ 30% vs. <30% | 59% | 41% | 34.5 | 18.2 | 16.3 | 0.52 (0.38-0.70) | <0.001 |
| | | FOLFOX-4 | 221 | ETS ≥ 30% vs. <30% | 38% | 62% | 30.7 | 16 | 14.7 | 0.46 (0.34-0.63) | <0.001 |
| Chemo. + anti-EGFR/VEGF in 1st line | FIRE-3 with ETS criterion 20% | FOLFIRI + Cet. | 157 | ETS ≥ 20% vs. <20% | 68% | 32% | 38.3 | 20.5 | 17.8 | 0.52 (0.34-0.80) | 0.002 |
| | | FOLFIRI + Bev. | 173 | ETS ≥ 20% vs. <20% | 49% | 51% | 31.9 | 21.2 | 10.7 | 0.49 (0.35-0.71) | <0.001 |
| | PEAK with ETS criterion 20% | mFOLFOX6 + Pani. | 80 | ETS ≥ 20% vs. <20% | 75% | 20% | 43.4 | 21.2 | 22.2 | 0.38 (0.20-0.69) | <0.001 |
| | | mFOLFOX6 + Bev. | 74 | ETS ≥ 20% vs. <20% | 62% | 28% | 32.5 | 21.8 | 10.7 | 0.42 (0.24-0.75) | <0.001 |
| | PEAK with ETS criterion 30% | mFOLFOX6 + Pani. | 80 | ETS ≥ 30% vs. <30% | 64% | 36% | 43.8 | 34.2 | 9.6 | 0.40 (0.22-0.72) | <0.001 |
| | | mFOLFOX6 + Bev. | 74 | ETS ≥ 30% vs. <30% | 45% | 55% | 35.1 | 23.9 | 11.2 | 0.43 (0.24-0.78) | <0.001 |
| | TRIBE with ETS criterion 20% | FOLFOXIRI + Bev. | 225 | ETS ≥ 20% vs. <20% | 63% | 30% | na | na | na | na | na |
| | | FOLFIRI + Bev. | 216 | ETS ≥ 20% vs. <20% | 52% | 40% | na | na | na | na | na |

Note: The p-values presented in the table were calculated via log-rank test and their numerical values were cited from the corresponding literatures.

**Supplementary discussion on comparison between DL-based and size-based criteria.** The comparison between DL and ETS criteria in the VELOUR trial as well as eight other well-known mCRC-related trials are summarized in Supplementary Table 1. The eight mCRC-related trials were found by conducting a literature search in the National Library of Medicine medical literature database via PubMed gateway with the key words 'early tumor shrinkage' or 'tumor size decrease' and 'metastatic colorectal cancer' (https://pubmed.ncbi.nlm.nih.gov/). The eight trails were BOND[1], 20050181 study [2], CRYSTAL [3], OPUS [3], PRIME [4], FIRE-3 [5], PEAK [6], and TRIBE [7], which including both phase II/III and first/second-line treatments. The data of the eight trails were cited from the corresponding published literatures. The comparison indicators included response rate (i.e., percentage of patients who responded to treatment), median OS and delta median OS (Δ), HR (95%CI), and p-value estimated by log-rank test.

Table 1 shows that our study was the first work to investigate early on-treatment prediction of outcome in mCRC patients receiving chemo. + anti-VEGF as a second-line treatment. In the VELOUR trial, the criteria "DL Score ≥ 0.6", "ETS ≥ 5%", and "ETS ≥ 20%" were all able to significantly stratify patients into responders and non-responders at the month-2 time point (p<0.001). Compared to the "ETS ≥ 5%", the "DL Score ≥ 0.6" showed superior performance in terms of a larger delta median OS, with 7.3 vs. 5.4 (Δ month) and 7.8 vs. 4.6 (Δ month) in the FA and F arms, respectively. Compared to the "ETS ≥ 20%", the "DL Score ≥ 0.6" showed superior performance in terms of higher response rate, with 46% vs. 25% and 37% vs. 11% in the FA and F arms respectively, and larger delta median OS, with 7.3 vs. 6.6 (Δ month) and 7.8 vs. 5.4 (Δ month) in the FA and F arms respectively as well.

Two comparison trials, the BOND and the 20050181, studied ETS in mCRC patients receiving chemo. + anti-VEGF as a 2nd line treatment. In the BOND trial, optimal ETS criterion "ETS ≥ 10%" was used rather than the "ETS ≥ 5%". In the 20050181 study, the general criterion "ETS ≥ 30%" was used. In terms of delta median OS, the performance of the "ETS ≥ 10%" in the BOND trial and the "ETS ≥ 5%" in the VELOUR trial were similar. But, in terms of response rate, both the BOND and 20050181 trials were much lower, especially the 20050181 trial which used the "ETS ≥ 30%". In studies of first-line chemo. + anti-VEGF treatment (*e.g.*, FIRE-3, PEAK and TRIBE), the ETS criteria "ETS ≥ 20%" and "ETS ≥ 30%" achieved higher response rate, with median and range of 66% (59% - 75%) and 49% (38% - 62%) in the experiment and control arms, respectively.

Overall, three trends of tumor size change patterns could be observed: 1) 1st line treatment introduced faster tumor shrinkage than 2nd line treatment; 2) anti-EGFR treatment introduced faster tumor shrinkage than anti-VEGF treatment; and 3) targeted therapy introduced faster tumor shrinkage than chemotherapy alone. Hence, it is a reasonable trend that, mCRC patients receiving chemo. + anti-VEGF as a 2nd line treatment showed a slow size change pattern in the VELOUR trail, which partially explained the superiority of DL-based criteria over ETS-based criteria.

# Supplementary Methods

## GoogLeNet Fine-tuning

The fine-tuning of GoogLeNet was implemented on Matlab version 9.5. The GoogLeNet we used was downloaded from https://www.mathworks.com/help/deeplearning/ref/googlenet.html. To fine-tune the GoogLeNet to domain of medical images, we created a dataset consisted of lesion images and tissue images. The lesion image set was regarded as positive set and contained totally 1000 lesion images randomly cropped from images of training cohort. Random rotation (range -30~30 degree) and re-scale (rang 0.7 ~1.5) were applied to augment the positive set to 5000 samples. Correspondingly, we constructed a negative set which contained 5000 tissue images randomly cropped from tissue regions. Thus, the downloaded pre-trained GoogLeNet was fine-tuned on the 10000 data set (5000 lesion images vs. 5000 tissue images) to differential lesions from tissues. The parameters for the fine-tuning were as follows,

```
options = trainingOptions('adam', ...
    'MaxEpochs',20, ...
    'InitialLearnRate', 0.0005, ...
    'LearnRateSchedule','piecewise', ...
    'LearnRateDropPeriod',5, ...
    'LearnRateDropFactor',0.95, ...
    'Shuffle','every-epoch'}.
```

Parameters that were not specified were set as default.

The layer for feature extraction was the 'pool5-7x7_s1', as indicated by the red arrow in Supplementary Figure 9.

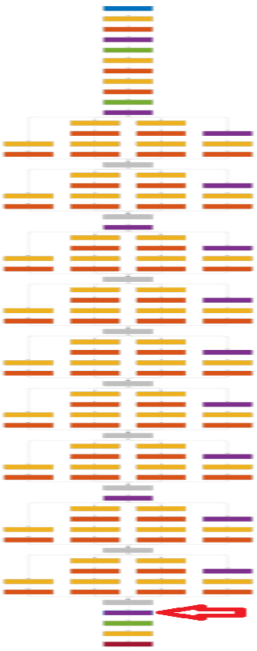

**Supplementary Figure 9. The network structure of GoogLeNet.** The red arrow indicates where the layer used for feature extraction is. To generate the structure picture in matlab, firstly, install GoogLeNet via the 'Add-on' application; then, load the GoogLeNet into the workspace; and finally, open the GoogLeNet using the 'deepNetworkDesigner' command.

## ROI preparation

Four image preprocessing procedures were applied for preparing inputs to DL networks. They were, 1) image spatial normalization, 2) image intensity normalization, 3) determination of region of interest (ROI), and 4) spatial augmentation of ROIs. Some details are as follows.

Firstly, due to the variability in slice thickness and in-plane resolution, all CT images were harmonized to homogenous voxel spacing of 1.0x1.0x1.0 $mm^3$ by using tri-linear interpolation. Secondly, since lesions in mCRC patients involved multi-organs, cropped ROI images were normalized by using CT window-level. Thirdly, to reduce the computation complexity, image ROIs containing lesions were cropped from the CT images to be as the inputs of network. The ROI of lesion was defined as a box of size 2*d* x 2*d* (where d is the length of diameter measured at baseline) with the center point corresponding to the center of the measurement line at baseline CT scan. It is noted that, lesion ROI at follow-up scan used the same *d* as that at baseline scan. Fourthly, to improve the robustness of the ROIs, in-plane spacing augmentations to ROIs were introduced, including random rotation (-30°~30°), shifting (-0.05*d*~0.05*d*) and scaling (0.95*d*~1.05*d*). The spatial augmentation for training data is ten folds. The selection of augmentation parameters was based on previous study that variability of tumor measurements in mCRC was about 10% [8].

After the image preprocessing, all ROI images were resized to the standard input size for the GoogLeNet, i.e., 224x224 pixel. An example for input of DL network training was presented in Supplementary Figure 10.

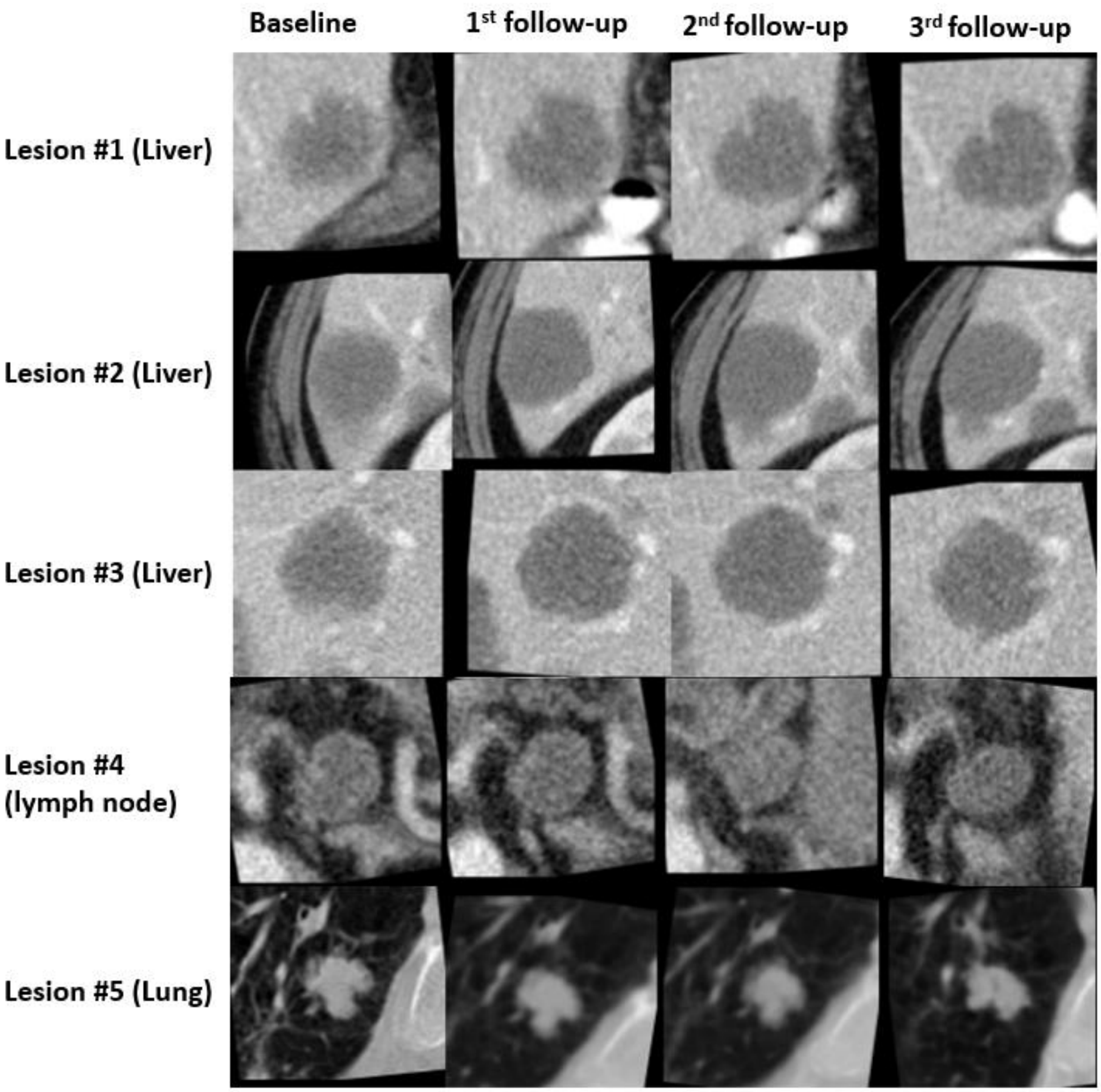


**Supplementary Figure 10. An example for input of DL network on training.** Five target lesions were selected, including three liver lesions, one lymph node lesion and one lung lesion. The CT scans were collected for the patient, including the baseline and the first three follow-ups.

## RNN construction and training

The construction and training of RNN were implemented on Matlab version 9.5. The code was available at https://drive.google.com/drive/folders/1LNULttbs9OzjeMzAiY0H705SdveJ175j?usp=sharing

## Reproducibility analysis

Two sets of DL prediction scores are compared. The two sets of DL prediction scores are attained via introducing random rotation (-30°~30°), shifting (-0.05*d*~0.05*d*) and scaling (0.95*d*~1.05*d*) to the original ROI. As mentioned in the 'ROI preparation' section above, the selection of augmentation parameters was based on previous study that variability of tumor measurements in mCRC was about 10% [8].

The comparison between the two sets of DL prediction scores is evaluated by the concordance correlation coefficient (CCC) [9].

# Supplementary References


1. Piessevaux, H., et al., *Radiological tumor size decrease at week 6 is a potent predictor of outcome in chemorefractory metastatic colorectal cancer treated with cetuximab (BOND trial).* Ann Oncol, 2009. **20**(8): p. 1375-82.
2. Peeters, M., et al., *546P Tumour Shrinkage And Response Outcomes During Second-Line Panitumumab (PMAB)+ Folfiri VS Folfiri Treatment.* J Annals of Oncology, 2014. **25**(suppl_4): p. iv186-iv187.
3. Piessevaux, H., et al., *Use of early tumor shrinkage to predict long-term outcome in metastatic colorectal cancer treated with cetuximab.* J Clin Oncol, 2013. **31**(30): p. 3764-75.
4. Douillard, J.Y., et al., *Impact of early tumour shrinkage and resection on outcomes in patients with wild-type RAS metastatic colorectal cancer.* Eur J Cancer, 2015. **51**(10): p. 1231-42.
5. Stintzing, S., et al., *FOLFIRI plus cetuximab versus FOLFIRI plus bevacizumab for metastatic colorectal cancer (FIRE-3): a post-hoc analysis of tumour dynamics in the final RAS wild-type subgroup of this randomised open-label phase 3 trial.* Lancet Oncol, 2016. **17**(10): p. 1426-1434.
6. Rivera, F., et al., *Final analysis of the randomised PEAK trial: overall survival and tumour responses during first-line treatment with mFOLFOX6 plus either panitumumab or bevacizumab in patients with metastatic colorectal carcinoma.* International journal of colorectal disease, 2017. **32**(8): p. 1179-1190.
7. Cremolini, C., et al., *Early tumor shrinkage and depth of response predict long-term outcome in metastatic colorectal cancer patients treated with first-line chemotherapy plus bevacizumab: results from phase III TRIBE trial by the Gruppo Oncologico del Nord Ovest.* Ann Oncol, 2015. **26**(6): p. 1188-94.
8. Zhao, B., et al., *Variability in assessing treatment response: metastatic colorectal cancer as a paradigm.* Clin Cancer Res, 2014. **20**(13): p. 3560-8.
9. Lin, L.I., *A concordance correlation coefficient to evaluate reproducibility.* Biometrics, 1989. **45**(1): p. 255-68.
