# Supplementary material for: Deep learning for the prediction of early on-treatment response in metastatic colorectal cancer from serial medical imaging
Source: Nat Commun. 2021 Nov 17;12:6654. doi: 10.1038/s41467-021-26990-6 (PMC8599694; doi:10.1038/s41467-021-26990-6)
Supplement: Supplementary file 2 — Reporting summary. [file 41467_2021_26990_MOESM2_ESM.pdf]

nature portfolio

Corresponding author(s): Lawrence H. Schwartz

Last updated by author(s): Oct 18, 2021

Reporting Summary

Nature Portfolio wishes to improve the reproducibility of the work that we publish. This form provides structure for consistency and transparency in reporting. For further information on Nature Portfolio policies, see our Editorial Policies and the Editorial Policy Checklist.

Statistics

For all statistical analyses, confirm that the following items are present in the figure legend, table legend, main text, or Methods section.

n/a	Confirmed	
[ ]	[x]	The exact sample size (*n*) for each experimental group/condition, given as a discrete number and unit of measurement
[x]	[ ]	A statement on whether measurements were taken from distinct samples or whether the same sample was measured repeatedly
[ ]	[x]	The statistical test(s) used AND whether they are one- or two-sided<br>*Only common tests should be described solely by name; describe more complex techniques in the Methods section.*
[x]	[ ]	A description of all covariates tested
[x]	[ ]	A description of any assumptions or corrections, such as tests of normality and adjustment for multiple comparisons
[ ]	[x]	A full description of the statistical parameters including central tendency (e.g. means) or other basic estimates (e.g. regression coefficient) AND variation (e.g. standard deviation) or associated estimates of uncertainty (e.g. confidence intervals)
[ ]	[x]	For null hypothesis testing, the test statistic (e.g. *F*, *t*, *r*) with confidence intervals, effect sizes, degrees of freedom and *P* value noted<br>*Give P values as exact values whenever suitable.*
[x]	[ ]	For Bayesian analysis, information on the choice of priors and Markov chain Monte Carlo settings
[x]	[ ]	For hierarchical and complex designs, identification of the appropriate level for tests and full reporting of outcomes
[x]	[ ]	Estimates of effect sizes (e.g. Cohen's *d*, Pearson's *r*), indicating how they were calculated

Our web collection on statistics for biologists contains articles on many of the points above.

Software and code

Policy information about availability of computer code

Data collection	Matlab version 9.5
Data analysis	Matlab version 9.5, R version 4.0.1, MedCalc version 15.8 and Microsoft Excel 2019

For manuscripts utilizing custom algorithms or software that are central to the research but not yet described in published literature, software must be made available to editors and reviewers. We strongly encourage code deposition in a community repository (e.g. GitHub). See the Nature Portfolio guidelines for submitting code & software for further information.

Data

Policy information about availability of data

All manuscripts must include a data availability statement. This statement should provide the following information, where applicable:

- Accession codes, unique identifiers, or web links for publicly available datasets
- A description of any restrictions on data availability
- For clinical datasets or third party data, please ensure that the statement adheres to our policy

The data that support the findings of this study are provided by the Vol-PACT (Advanced Metrics and Modeling with Volumetric Computed Tomography for Precision Analysis of Clinical Trial Results), which is a program under the Foundation for the National Institutes of Health (FNIH) Biomarkers Consortium (https://fnih.org/our-programs/biomarkers-consortium/programs/vol-pact). Since the fNIH Biomarkers Consortium is a public–private partnership, the data is available with permission from the Foundations, Biomarker Consortium. That is, to access the data, companies and not-for-profit organizations should firstly become a member of Biomarkers Consortium and then participate the Vol-PACT. The information on joining Biomarker Consortium can be found at, https://fnih.org/our-programs/biomarkers-consortium/join. And the application for participating the Vol-PACT can contact the program manager of Vol-PACT at the Biomarker Consortium, Dana E. Connors (Senior Scientific Program Manager, Cancer Research Partnerships) at dconnors@fnih.org.

Field-specific reporting

Please select the one below that is the best fit for your research. If you are not sure, read the appropriate sections before making your selection.

- [x] Life sciences
- [ ] Behavioural & social sciences
- [ ] Ecological, evolutionary & environmental sciences

For a reference copy of the document with all sections, see nature.com/documents/nr-reporting-summary-flat.pdf

Life sciences study design

All studies must disclose on these points even when the disclosure is negative.

Sample size	Since our study is a re-analysis of a completed trail, details of the trial, including ethical regulation, trail approval, patient eligibility, trial design, patient randomization, and dose administration, can be found in the previous report, 'Van Cutsem, E., et al., Addition of aflibercept to fluorouracil, leucovorin, and irinotecan improves survival in a phase III randomized trial in patients with metastatic colorectal cancer previously treated with an oxaliplatin-based regimen. J Clin Oncol, 2012. 30(28): p. 3499-506'.
Data exclusions	Since our study is a re-analysis of a completed trail, details of the trial, including ethical regulation, trail approval, patient eligibility, trial design, patient randomization, and dose administration, can be found in the previous report, 'Van Cutsem, E., et al., Addition of aflibercept to fluorouracil, leucovorin, and irinotecan improves survival in a phase III randomized trial in patients with metastatic colorectal cancer previously treated with an oxaliplatin-based regimen. J Clin Oncol, 2012. 30(28): p. 3499-506'.
Replication	Since our study is a re-analysis of a completed trail, details of the trial, including ethical regulation, trail approval, patient eligibility, trial design, patient randomization, and dose administration, can be found in the previous report, 'Van Cutsem, E., et al., Addition of aflibercept to fluorouracil, leucovorin, and irinotecan improves survival in a phase III randomized trial in patients with metastatic colorectal cancer previously treated with an oxaliplatin-based regimen. J Clin Oncol, 2012. 30(28): p. 3499-506'.
Randomization	Since our study is a re-analysis of a completed trail, details of the trial, including ethical regulation, trail approval, patient eligibility, trial design, patient randomization, and dose administration, can be found in the previous report, 'Van Cutsem, E., et al., Addition of aflibercept to fluorouracil, leucovorin, and irinotecan improves survival in a phase III randomized trial in patients with metastatic colorectal cancer previously treated with an oxaliplatin-based regimen. J Clin Oncol, 2012. 30(28): p. 3499-506'.
Blinding	Since our study is a re-analysis of a completed trail, details of the trial, including ethical regulation, trail approval, patient eligibility, trial design, patient randomization, and dose administration, can be found in the previous report, 'Van Cutsem, E., et al., Addition of aflibercept to fluorouracil, leucovorin, and irinotecan improves survival in a phase III randomized trial in patients with metastatic colorectal cancer previously treated with an oxaliplatin-based regimen. J Clin Oncol, 2012. 30(28): p. 3499-506'.

Reporting for specific materials, systems and methods

We require information from authors about some types of materials, experimental systems and methods used in many studies. Here, indicate whether each material, system or method listed is relevant to your study. If you are not sure if a list item applies to your research, read the appropriate section before selecting a response.

Materials & experimental systems

n/a	Involved in the study	
x		Antibodies
x		Eukaryotic cell lines
x		Palaeontology and archaeology
x		Animals and other organisms
x		Human research participants
	x	Clinical data
x		Dual use research of concern

Methods

n/a	Involved in the study	
x		ChIP-seq
x		Flow cytometry
x		MRI-based neuroimaging

Clinical data

Policy information about clinical studies

All manuscripts should comply with the ICMJEguidelines for publication of clinical research and a completedCONSORT checklist must be included with all submissions.

Clinical trial registration	NCT00561470
Study protocol	https://clinicaltrials.gov/ct2/show/NCT00561470
Data collection	Since our study is a re-analysis of a completed trail, details of the trial, including ethical regulation, trail approval, patient eligibility, trial design, patient randomization, and dose administration, can be found in the previous report, 'Van Cutsem, E., et al., Addition of aflibercept to fluorouracil, leucovorin, and irinotecan improves survival in a phase III randomized trial in patients with metastatic colorectal cancer previously treated with an oxaliplatin-based regimen. J Clin Oncol, 2012. 30(28): p. 3499-506'.
Outcomes	Since our study is a re-analysis of a completed trail, details of the trial, including ethical regulation, trail approval, patient eligibility, trial design, patient randomization, and dose administration, can be found in the previous report, 'Van Cutsem, E., et al., Addition of aflibercept to fluorouracil, leucovorin, and irinotecan improves survival in a phase III randomized trial in patients with metastatic colorectal cancer previously treated with an oxaliplatin-based regimen. J Clin Oncol, 2012. 30(28): p. 3499-506'.
